# Electromechanical tensile test equipment for stretchable conductive materials

**DOI:** 10.1016/j.ohx.2022.e00287

**Published:** 2022-03-07

**Authors:** Ardi Wiranata, Yunosuke Ohsugi, Ayato Minaminosono, Yu Kuwajima, Shingo Maeda

**Affiliations:** a Smart Materials Laboratory, Department of Engineering Science and Mechanics, Shibaura Institute of Technology, 3-7-5, Toyosu, Koto City, Tokyo 135-8548, Japan; b Department of Mechanical and Industrial Engineering, Universitas Gadjah Mada, Jalan Grafika No. 2, Yogyakarta 55281, Indonesia

**Keywords:** Conductive elastomer, Electromechanical properties, Soft robotics, Soft sensor, Tensile test

## Abstract

The demand for soft and conductive materials has intensified due to the increased interest in soft robotics. Consequently, researchers strive to realize easy, fast, and cost-effective fabrication methods. To evaluate the mechanical properties of materials requires tensile testing. However, the availability of an electromechanical tensile test to assess the quality of the electromechanical properties of stretchable conductive materials has yet to be widely commercialized. This situation has hindered the development of soft and stretchable conductive materials. Here, we develop a customized electromechanical tensile test for soft and stretchable materials. We integrate three standalone devices using Python software and provide a graphic user interface (GUI) for easy operation of the equipment. We expect that our customized electromechanical tensile test will contribute to advances in soft robotics, especially soft and stretchable sensors. Furthermore, our electromechanical setup can aid in the development of laboratory equipment and the understanding of the electromechanical properties of stretchable conductive materials.

## Graphical abstract

**Figure:**
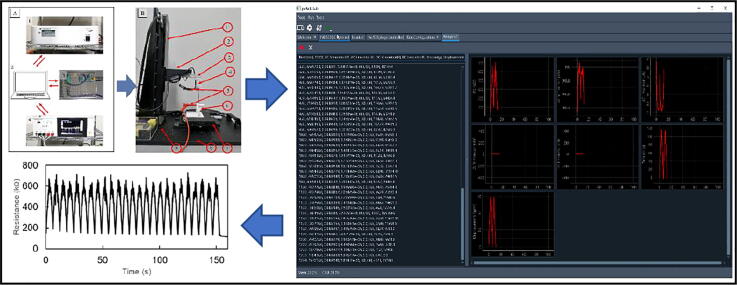

**Specifications table:** 

Hardware name	Electromechanical Tensile Test Equipment for Stretchable Conductive Materials
Subject area	Engineering and materials science
Hardware type	Measuring physical properties and in-lab sensors
Closest commercial analog	Tensile tester machine coupled with electrical properties of materials. Specialized for soft conductive material
Open source license	Creative Commons Attribution-ShareAlike 4.0 International License (CC BY-4.0)
Cost of hardware	$10,311
Source file repository	http://dx.doi.org/10.17632/rckgk7gz5m.1

## 1 Hardware in context

Sott robotics has received increased attention in different fields due to potential applications such as wearable devices [1], [2], [3], biomedical [4], [5], [6], healthcare [7], [8], [9], and other human soft machine interfaces. Fundamental research has been conducted to support advances in soft robotics. For example, studies on soft actuators (e.g., dielectric elastomer actuators (DEAs) [10], [11], [12], [13], soft electro-adhesion [14], [15], [16], [17], and stretchable pumps [18], [19], [20]) provide a basic understanding of their characteristics. The primary strategy for developing soft robotics involves innovation in soft materials engineering, including materials synthesis, fabrication, and mechanical design [21]. However, a major hurdle is that the whole body, including the electric circuit, must be bendable, twistable, and stretchable. Consequently, conductive and stretchable materials are crucial.

As the interest in soft robotics grows, the demand for flexible conductive materials has increased. Much effort has been devoted to discovering fast, reliable, and cost-effective fabrication methods for flexible conductive materials. For example, stretchable sensors have been formed using carbon black (CB) mixed with Ecoflex [22], 3D printing [23], and ionic liquids for human motion monitoring [24]. When fabricating stretchable sensors, multiple areas must be considered: mechanical compliance, mechanical characteristics, and electric conductivity of the material. Conductive materials that do not undergo physical changes or damage under cyclic tensile and high strain conditions should exhibit a high performance.

To investigate these characteristics, special equipment for stretchable sensor tests should be established. Several methods are employed to assess the quality of stretchable conductive materials. These include mechanical testing, electrical properties testing, and physical inspection. Physical inspection elucidates how a stretchable material can be conductive. For example, DEAs can be fabricated via a brushing method of carbon nanotube (CNT) powders [10]. Researchers use commercially available field emission scanning electron microscopes to understand how brushing affects the DEA quality. This physical inspection is performed easily since the equipment is available in the marketplace. On the other hand, testing the mechanical and electrical properties of stretchable conductive materials is more challenging since combined equipment that measures mechanical properties and electrical properties (electromechanical test) simultaneously is not widely available in the marketplace.

Previous studies have implicitly used an electromechanical equipment test to check the quality and characteristics of stretchable sensors. For example, CB-Ecoflex conductive material was examined using a motorized stage combined with an LCR meter [22]. They controlled the equipment through LabView to evaluate the electromechanical properties of the stretchable sensor. Another example is the development of stretchable sensors using brushing methods for DEAs [10]. They also used a tensile tester machine synchronized with an LCR meter to assess the condition of stretchable conductive materials. Both of these studies focused on physical phenomena of the soft sensor and actuators. Neither provided details about the electromechanical equipment, equipment synchronization, or codes for device synchronization. In this study, we establish clear steps and methods to setup electromechanical tensile test equipment for soft stretchable conductive materials. We provide Python-based software so that every researcher can access the software and modify it to meet their research requirements. This study should assist in the development of laboratory equipment and research.

## 2 Hardware description

**Fig. 1:**
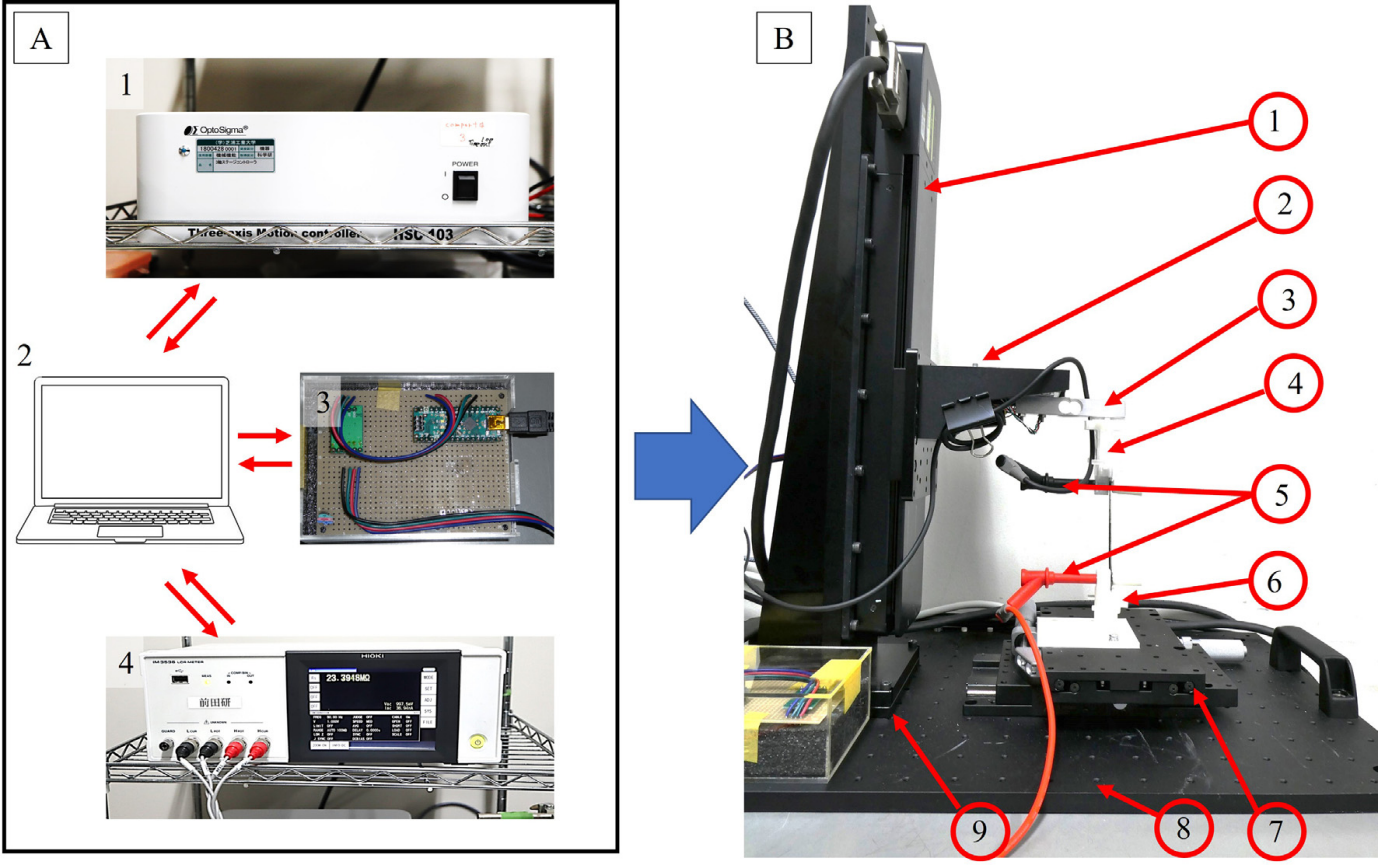
Electromechanical tensile test equipment. A. controller and data acquisition part, and B. testing part.

The described equipment aims to integrate a mechanical tensile test with an electrical property test. The equipment is composed of three modules: a linear stage and controller, an LCR meter, and a customized weight scale. All of these modules are integrated using a Python GUI interface for operation ease. Fig. 1 depicts the overall electromechanical tensile test device integration. Fig. 1A describes the controller and data acquisition part, while Fig. 1B shows the testing condition part. In general, the device is composed of three modules (tensile tester module, electrical tester module, and GUI). Details of each module are described below.

### 2.1 Tensile tester module

**Fig. 2:**
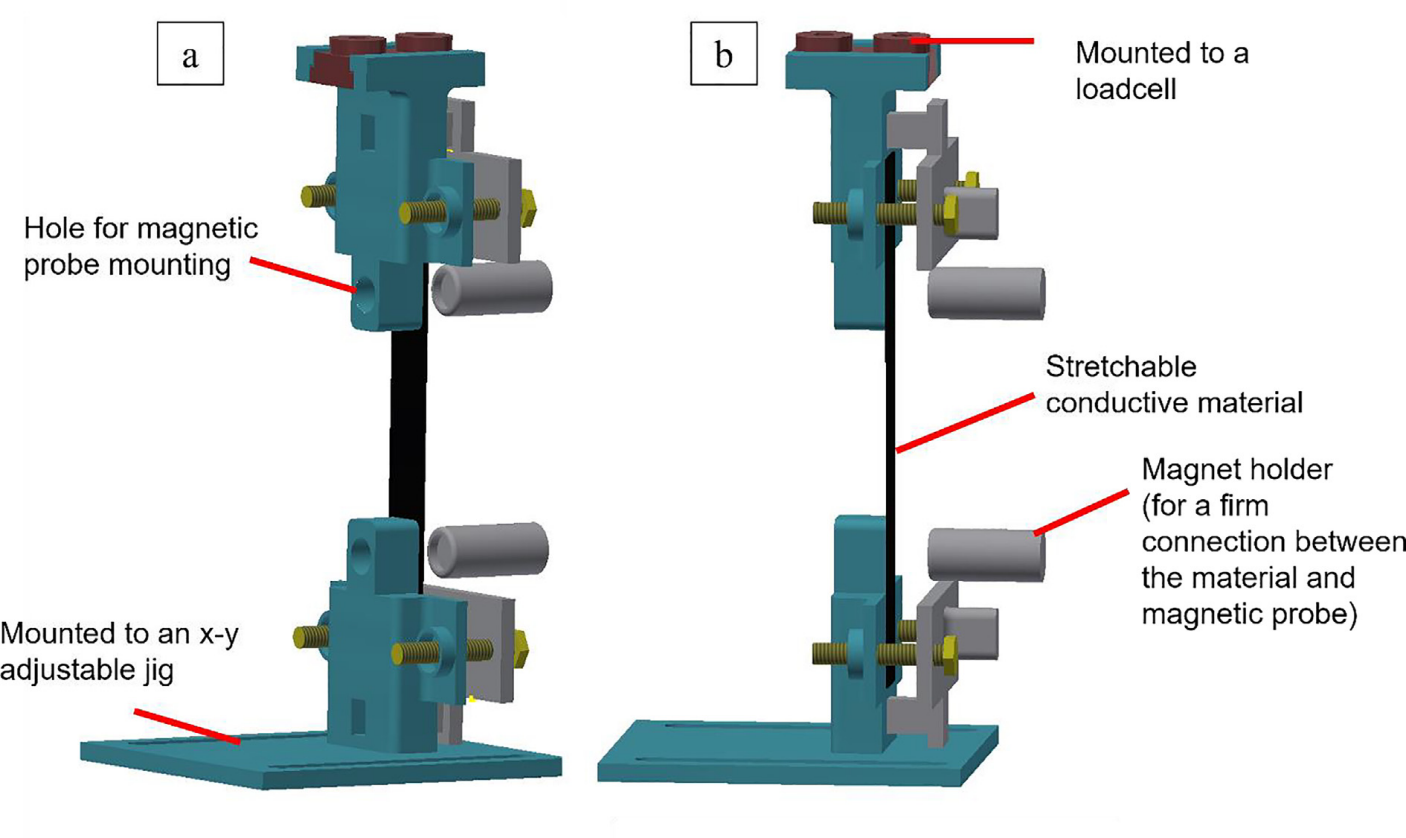
Material gripper for electromechanical tensile test equipment.

The tensile tester module consists of a linear stage controller by Optosigma HSC-103 (Fig. 1 A1), linear stage OSMS-26-300ZSGSP by Optosigma (Fig. 1 B1), manual adjustable X-Y jig by Optosigma (Fig. 1 B7), a loadcell with a maximum load of 5 kg by Uxcell (Fig. 1 B3) controlled by HX711 module with Arduino-Nano (Fig. 1 A3), and 3D-printed material gripper (Figs. 1 B4 and B6). The linear stage and the controller part are used without any modifications. We bought an X-Y adjustable jig by Optosigma separately to easily adjust the bottom part of the gripper in the x and y-direction. A commercially available loadcell module with Arduino-Nano is employed to easily integrate all of the devices with the GUI since Arduino-Nano can be controlled using serial communication. The trickiest part in this module is designing the gripper (Figs. 1 B4 and B6). The gripper part should accommodate the material gripper and the conductive probe to monitor the resistance change in the material. Herein we designed the gripper for resistance monitoring. Fig. 2 shows the material gripper part. The hole in Fig. 2a is to mount the HIOKI magnetic probe. We use a magnetic holder to secure the connection between the magnetic probe and the conductive material (Figs. 1B and 2b). Compared to other commercialized tensile test machines, the price of our device is reasonable and supports fully customized device arrangements for a specific purpose.

### 2.2 Electrical tester module

The electrical tester module consists of an LCR meter HIOKI 3536 (Fig. 1A4) and a magnetic probe HIOKI 9804 (Fig. 1B5). For an electromechanical test, the magnetic probe is inserted in the gripper mount (Fig. 2a). The LCR meter has a universal serial bus (USB) serial support for communication between the LCR meter and a personal computer (PC). This allows the equipment to be controlled using simple Python code.

### 2.3 GUI of the tensile tester

**Fig. 3:**
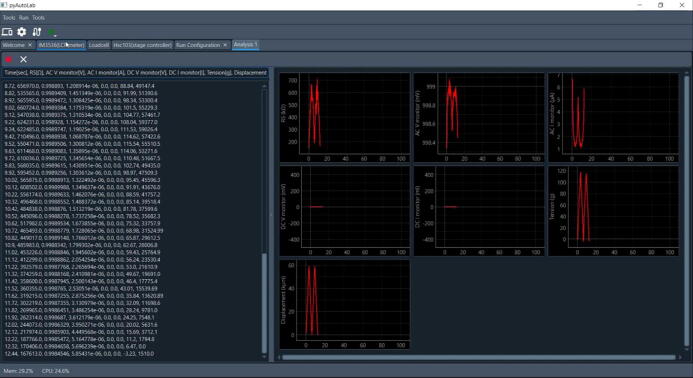
GUI of the tensile test equipment.

In this study, the GUI helps researchers conduct the experiment by easily defining the appropriate variable. We created the GUI with a Python platform. Fig. 3 shows the overall GUI when a measurement is in progress. As this software is an opensource software, researchers can easily access and develop the software based on their research objective. The chart monitoring side in this GUI shows some information, for example, resistance, voltage, current, displacement of the linear stage, and tension. In this GUI, the tension and the displacement are in grams and millimeters, respectively. The strain and tensile strength can be manually converted from the raw data using spreadsheet software.

The goal of this system integration is to measure the mechanical and electrical properties simultaneously. This experimental setup may contribute to the development of soft and stretchable conductive materials for soft sensors, soft actuators, and soft electric circuits. Our fabrication approach is useful for researchers interested in the following subjects:

• Flexible electronics: An electric circuit must be composed of a flexible material that is conductive even under high strain conditions. This electromechanical tensile test can help elucidate the basic qualities of a stretchable conductive material under strained conditions.
• Soft robotics: High-performance stretchable sensors are necessary to provide an interface between human and soft robotics. In addition, this electromechanical testing device could help researchers understand the basic performance characteristics of stretchable sensors.
• Soft materials engineering: The tensile test equipment can perform electrical and mechanical tests simultaneously. This equipment is suitable for soft material tensile tests. This device also can help characterize polymer or rubber (cyclic and one-time tensile tests).
• Customized laboratory experiments: The equipment is commercially available in the marketplace and sold separately. Any researcher can setup this tensile testing equipment. The price is reasonable compared to other professional packs of tensile tester machines. Moreover, the equipment can be customized and arranged based on the user’s requirements.

## 3 Design file summary

**Table 1:** Design file name and summary.

Design file name	File type	Opensource license	Location of the file
Gripper (magnetic holder, upper side, and bottom side)	CAD files	CC BY-4.0	(Folder: Gripper 3D file) http://dx.doi.org/10.17632/rckgk7gz5m.1
Python GUI software interface	Python program files	CC BY-4.0	(Folder: Software GUI (python software)) http://dx.doi.org/10.17632/rckgk7gz5m.1
Arduino Program for loadcell	Arduino program	CC BY-4.0	(Folder: Loadcell-Arduino Software) http://dx.doi.org/10.17632/rckgk7gz5m.1
Equipment Installation process	Word file	CC BY-4.0	(Folder: Driver list) http://dx.doi.org/10.17632/rckgk7gz5m.1
GUI software setting movie (Movie S1)		CC BY-4.0	(Folder: Movie S1) http://dx.doi.org/10.17632/rckgk7gz5m.1

## 4 Bill of materials

**Table 2:** Bill of materials.

Designator	Component	Number	Cost per unit -currency	Total cost -currency	Source of materials	Material type
Linear stage (Fig. 1B1)	OSMS-26-300(Z) SGSP	1	$1,765	$1,765	https://jp.optosigma.com/	Metal
Linear stage controller (Fig. 1A1)	HSC-103	1	$2,621	$2,621	https://jp.optosigma.com/	Metal
Arduino-Nano (Fig. 1A3)	Arduino-Nano	1	$25	$25	https://www.amazon.co.jp/	Electronics
LCR meter (Fig. 1A4)	IM3536	1	$4,194	$4,194	https://www.hioki.com	Electronics
Z bracket (Fig. 1B2)	ZBR-8060	1	$34	$34	https://jp.optosigma.com/	Metal
Loadcell 5 kg and HX711 from Uxcell (Fig. 1B3)	Loadcell & HX711	1	$12	$12	https://www.amazon.co.jp/	Metal and Electronics
Gripper (Figs. 1B4 and B6)	magnetic holder, upper side, and bottom side	1	$3	$3	3Dprinted part	Polylactic acid (PLA) (3D printed material)
Magnetic probe (Fig. 1B5)	HIOKI 9804	1	$58	$58	https://www.amazon.co.jp/	Meral and plastic
X-Y jig (Fig. 1B7)	TSD-10162SR	1	$1,310	$1,310	https://jp.optosigma.com/	Metal
Breadboard (Fig. 1B8)	OBC-4545	1	$282	$282	https://jp.optosigma.com/	Metal
Conversion spacer (Fig. 1B9)	SP-102–2	1	$9	$9	https://jp.optosigma.com/	Metal

## 5 Build instructions

The assembly process of the tensile test equipment requires the following parts: linear stage, linear stage controller, Z bracket, breadboard, conversion spacer, X-Y jig, and 3D printed gripper. We purchased all these parts and used them without any modifications. We designed the gripping part for soft material tensile testing using an Autodesk Inventor for students and printed it using a 3D printer (3D raise Pro 2). Since all parts are used as received, the assembly process is straightforward with the following steps. First, bolt the conversion spacer onto the breadboard. Second, bolt the linear stage onto the conversion spacer. Third, bolt the X-Y jig onto the breadboard exactly in front of the linear stage, as shown in Fig. 1B. Fourth, place the Z bracket onto the linear stage at the moving stage. At this point, our tensile tester is almost ready. To complete the equipment, bolt the loadcell to the Z bracket. Finally install the upper gripper on the loadcell and the lower gripper on the X-Y jig. Now the tensile tester part is complete.

To control the tensile tester part and acquire data for both tensile displacement and tensile strength, connect the linear stage to the linear stage controller (HSC-103). Then connect HSC-103 to a PC via a USB port. Now the linear stage can be controlled using our Python GUI (Fig. 3). Next, activate the loadcell reading by connecting the loadcell with HX711 and HX711 with Arduino-Nano. This loadcell module is a do-it-yourself (DIY) kit, which is already supported with the Arduino library. Hence, installation is straightforward. First, connect the loadcell wire to HX711 with the connection chart in Table 3. Then connect HX711 to Arduino-Nano according to Table 3. To acquire tensile strength data, connect Arduino-Nano to a personal computer and read the data using our Python GUI. This completes the tensile tester assembly.

To acquire electrical data, insert the magnetic probe (HIOKI 9804) into the hole provided in the upper and bottom sides of the 3D-printed gripper. The magnetic probe should be aligned with the gripper surface. Next, connect the magnetic probe to the LCR meter. Connect the LCR meter to the computer. This completes the electromechanical tensile tester assembly. Finally, connect Arduino-Nano, LCR meter, and linear stage controller to the computer and launch the provided Python GUI to control and read the data. Fig. 1 shows the final equipment arrangement.

## 6 Operation instructions

The provided Python GUI can control the above assembly. First, install the required drivers provided by the company of each part. The detailed process on the driver installation is available in the following repository as shown in Table 1 (Driver List). Next, install the loadcell module. The loadcell module is an Arduino-based module, which makes installation straightforward. Briefly, the installation of the loadcell module begins by installing the Arduino ide software and the HX711 library. Afterwards, upload the provided program to the Arduino board. Details about the software upload and installation of the Arduino program are available in the repository in Table 1 (Driver List folder and the arduino program for loadcell provided in folder Loadcell-Arduino Software).

**Table 3:** Connection chart of the loadcell module.

Load cell to HX711	
Loadcell side (Cable color)	HX711 (Pin name)
Red	E+
Black	E-
White	A-
Green	A+

**HX711 to Arduino-Nano**	

**HX711** **(Pin name)**	**Arduino-Nano** **(Pin name)**

GND	GND
DT	A1
SCK	A0
VCC	5 V

**Fig. 4:**
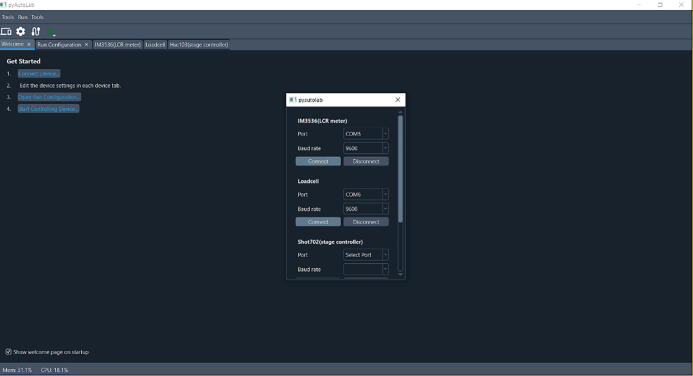
Home screen of the electromechanical device GUI control.

After the installation process is finished, control can be realized from our Python GUI. Our Python GUI sends the commands provided by the vendor of each part. In the LCR meter, the vendor provides command codes to get the data and so for HSC-103. In the case of the loadcell module, we make our command since the program to read the load cell is simple. In the loadcell module, we use command codes “a” and “b” for data reading and tare scaling, respectively. Then equipment control begins by installing necessary Python libraries as mentioned in the repository in Table 1 (folder name is Software GUI (python software)). After installing the essential Python library, the GUI is started by running the start.py file (all of the program instructions are in the repository in Table 1 (folder name is Software GUI (python software)). After the program is opened, the connection ports must be set (Fig. 4). Choose the port for the LCR meter, HSC 103, and Loadcell module. Then click connect to each tab and close this step. The GUI provides control for two linear stage controllers: HSC 103 and SHOT 702. These two controllers are also compatible with OSMS 26 300(Z). Thus, SHOT 702 or HSC 103 can be used to control OSMS 26 300(Z).

**Fig. 5:**
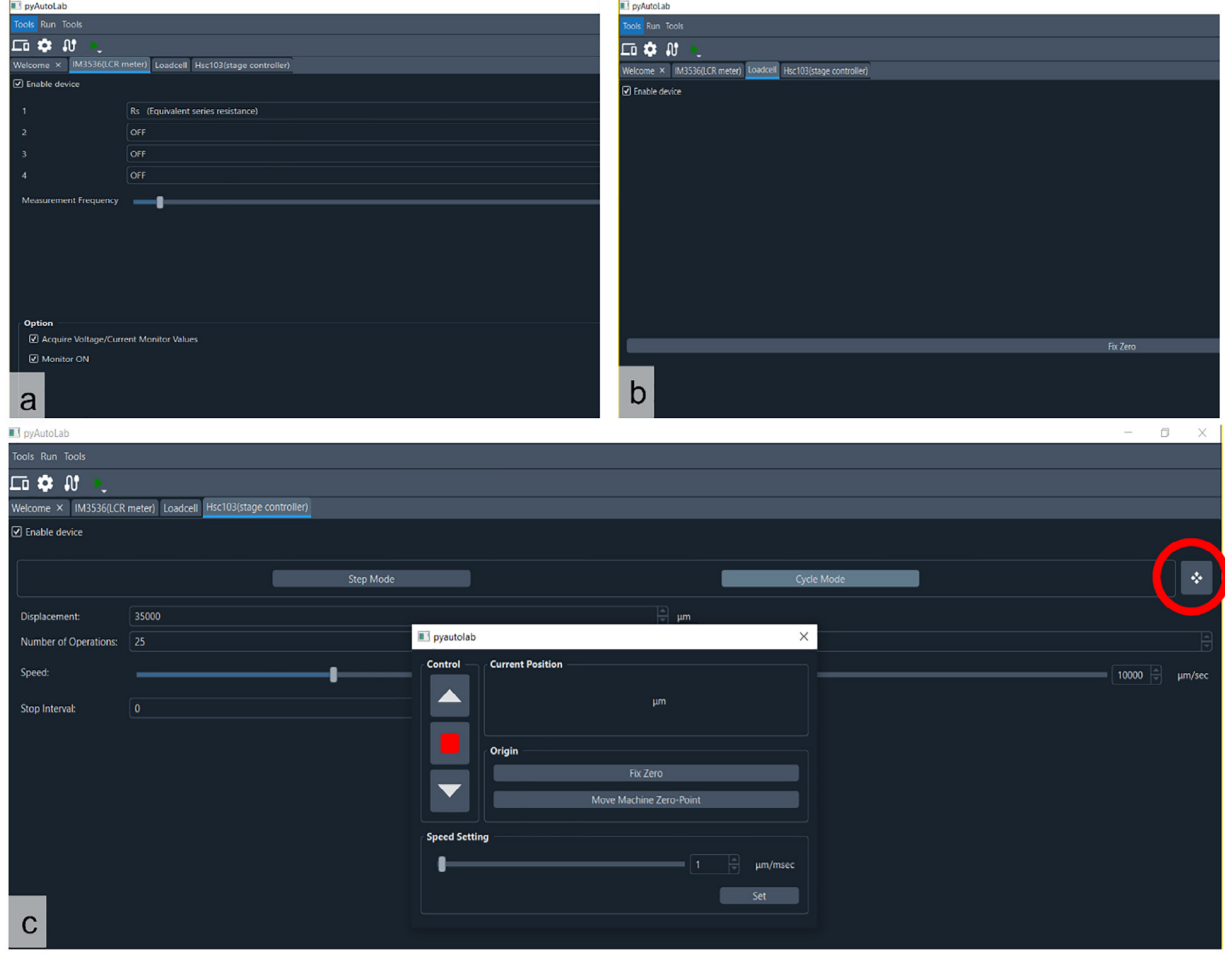
Setup of the variable for testing equipment. A. LCR meter tab, b. loadcell tab, and c. linear stage control tab.

**Fig. 6:**
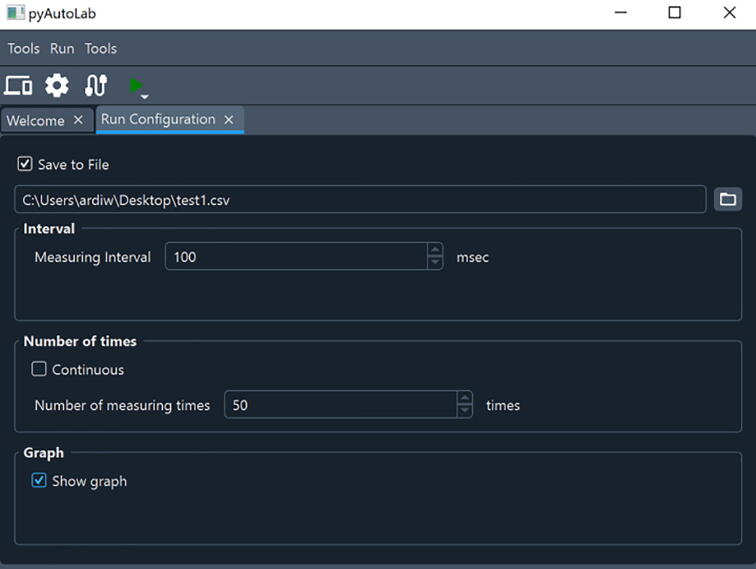
Run configuration option.

Fig. 5 shows the equipment setting tab, start from the LCR meter (Fig. 5a), loadcell (Fig. 5b), and linear stage controller tab (Fig. 5c). In the LCR meter tab, many variables can be selected. Start from resistance or any other variable such as capacitance or impedance. Four variables can be chosen simultaneously by selecting 4 in the dropdown menu. The loadcell tab is simple since it can include or exclude the equipment by checking or unchecking the box to enable device, respectively. The fix zero button is used to tare the scale (i.e., set the current weight as a reference). Then at the linear stage tab, first, set the zero position of the linear stage by clicking the red circled button (Fig. 5c). Then a new window will appear, as shown in Fig. 5c. The position can be adjusted and then click zero to set the current position to zero. Then decide whether the testing method is one step, many stepping modes of a one-time tensile test, or a cyclic tensile test. Next, determine the maximum displacement of the tensile test and the number of cycles (if it is a cyclic test). If there is an interval between cycles, add this parameter. Furthermore, set the pulling speed of the tensile tester. In the next steps, we navigate to the welcome tab again (without closing any of the tabs) and click the “open run configuration” (Fig. 6). In the run configuration, define the location to save the raw data, the measuring interval, and whether to show the graphic in real-time. Once these settings are finished, navigate to the welcome tab again and click final step number four (Fig. 4) to start controlling the device or click the green triangle-shaped button at the menu bar. (Movie S1 shows the detailed setup process. Movie S1 is available in repository in Table 1.

## 7 Validation and characterization

**Fig. 7:**
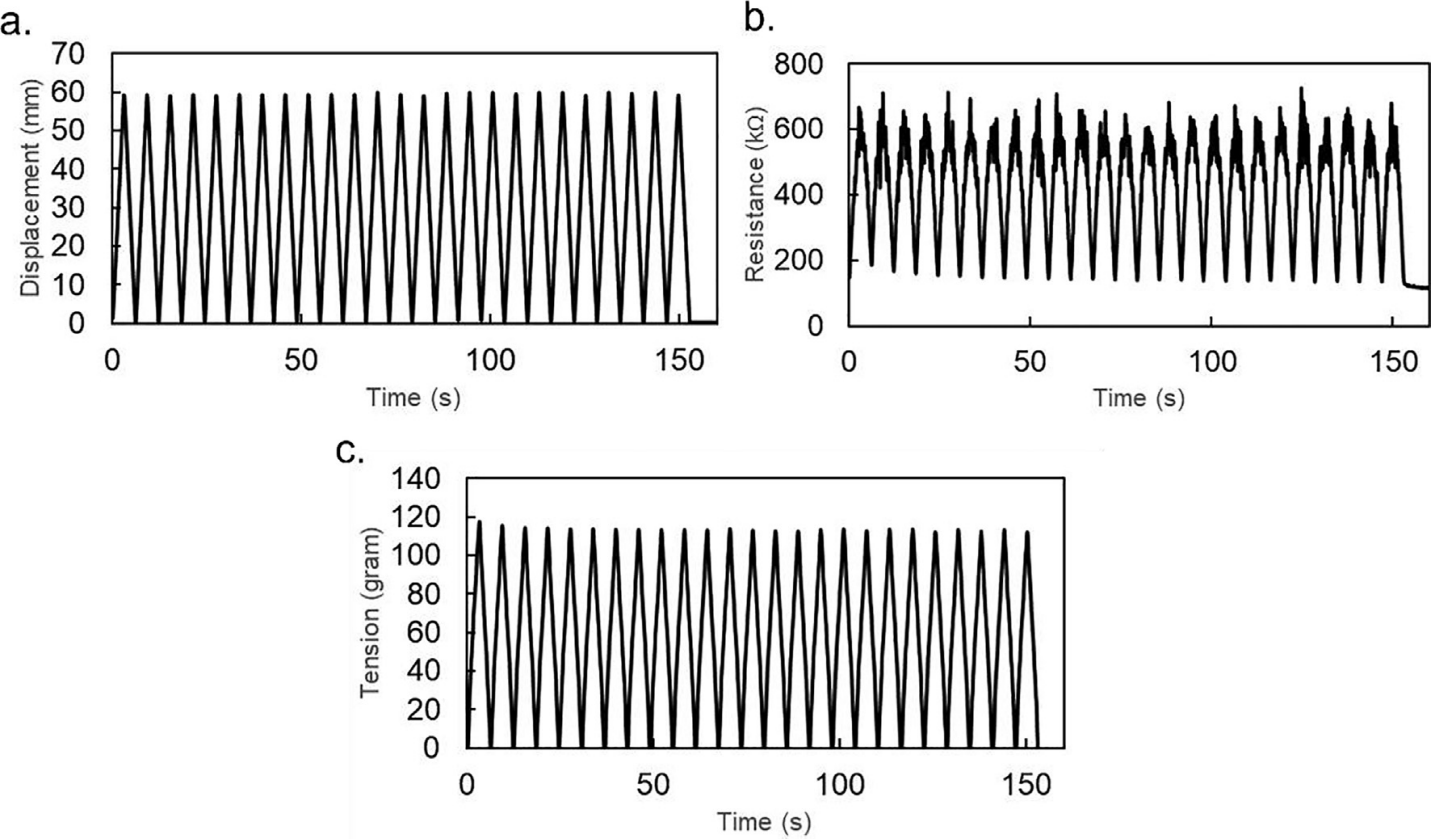
Raw data acquired from the electromechanical tensile test data.

Using the setup in the previous section, this chapter demonstrates an electromechanical tensile test for testing stretchable conductive materials. The experimental setup is shown in Fig. 1B. We previously tested our equipment for hundreds of cyclic tests of stretchable conductive elastomers and found that the most stable operation settings are achieved with a measuring interval of 100 ms (around 10 Hz of data transfer). We also tested the equipment at different tensile speeds ranging from 10 to 30 mm/s. The measurement process is stable. In this section, we show data acquired from actual measurements. Fig. 7 shows the first 25 cyclic tensile tests of a conductive stretchable material acquired at a tensile speed of 10 mm/s and a sampling rate of 100 ms.

**Fig. 8:**
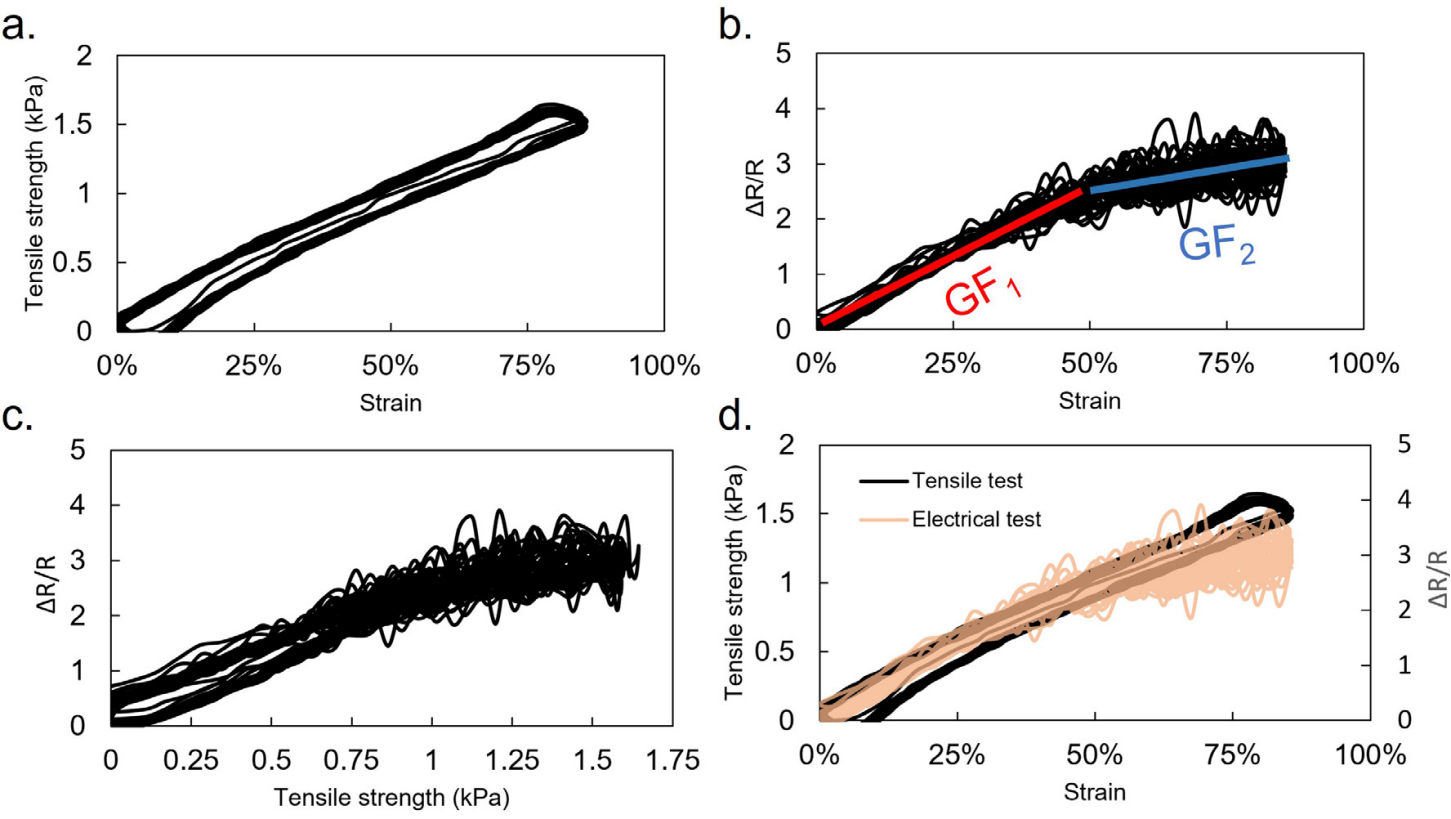
Processed data depicting the characteristics of a stretchable conductive material. a. Mechanical characteristics of the stretchable material, b. electric properties of the stretchable material, c. tensile strength change versus ΔR/R, and d. comparison between the mechanical and the electrical properties of the stretchable conductive material.

Since the data (Fig. 7) are acquired simultaneously, the characteristics of the stretchable conductive material can be extracted. Fig. 8 presents the processed data of the stretchable conductive material properties. An important electromechanical property for stretchable sensors is the gauge factor (GF). The GF resembles the sensitivity of the sensor due to strain changes. The higher the GF, the more sensitive the sensor. The GF value is calculated by dividing the resistance relative change (*ΔR/R*) and the strain change of the sensor (*ε*). From the sample data in Fig. 8b, we roughly estimate that GF_1_ is approximately 5 and GF_2_ is approximately 1.5. Because the data is acquired simultaneously, it is clear that the decrease in GF is not correlated with the mechanical properties of the sensor since the stress–strain data is linear but the GF value fluctuates. This kind of sample data may help researchers understand the characteristics of their stretchable conductive materials. We expect that our electromechanical test equipment will aid researchers in soft robotics by helping them setup experimental equipment easily.

## 8 Conclusion

This paper integrates equipment to test the electrical and mechanical properties. Simultaneously testing the mechanical and electrical properties should elucidate the essential characteristics of stretchable conductive materials. Although our equipment is designed specifically for soft materials, it could be used for hard materials such as PVC, carbon composites, or nylon by modifying the gripping part. Additionally, our equipment has the potential to measure the properties of touch and pressure sensors by modifying the gripping part. Changing the linear stage controller from HSC-103 to SHOT 702 should reduce the overall price since SHOT 702 is $1000 cheaper than HSC103. We expect that our equipment will contribute to the development of soft robotics, especially soft and stretchable sensors.

## Ethics statements

We confirmed that our work does not involve any animal or human experiments.

## CRediT authorship contribution statement

**Ardi Wiranata:** Conceptualization, Methodology, Writing – original draft. **Yunosuke Ohsugia:** Software, Data curation. **Ayato Minaminosonoa:** Visualization, Investigation. **Yu Kuwajima:** Software, Validation. **Shingo Maeda:** Supervision, Writing – review & editing.

## Declaration of Competing Interest

The authors declare that they have no known competing financial interests or personal relationships that could have appeared to influence the work reported in this paper.
